# KCNK levels are prognostic and diagnostic markers for hepatocellular carcinoma

**DOI:** 10.18632/aging.102311

**Published:** 2019-10-02

**Authors:** Wen-Chao Li, Zhi-Yong Xiong, Pin-Zhu Huang, Yang-Jing Liao, Quan-Xi Li, Zhi-Cheng Yao, Ya-Di Liao, Shi-Lei Xu, Hui Zhou, Qing-Liang Wang, He Huang, Peng Zhang, Ji-Zong Lin, Bo Liu, Jie Ren, Kun-Peng Hu

**Affiliations:** 1 Department of General Surgery, The Third Affiliated Hospital, Sun Yat-sen University, Guangzhou, China; 2 Guangdong Provincial Key Laboratory of Colorectal and Pelvic Floor Diseases, The Sixth Affiliated Hospital, Sun Yat-sen University, Guangzhou, China; 3 Department of Traditional Chinese Medicine, The Third Affiliated Hospital, Sun Yat-sen University, Guangzhou, China; 4 Department of Radiology, The Third Affiliated Hospital, Sun Yat-sen University, Guangzhou, China; 5 State Key Laboratory of Oncology in South China, Collaborative Innovation Center for Cancer Medicine, Sun Yat-sen University Cancer Center, Guangzhou, China; 6 Department of Ultrasound, The Third Affiliated Hospital of Sun Yat-sen University, Guangzhou, China

**Keywords:** hepatocellular carcinoma, KCNK, prognosis, TCGA, Kaplan-Meier plot

## Abstract

Two-pore-domain (KCNK, K2P) K^+^ channels are transmembrane protein complexes that control the flow of ions across biofilms, which underlie many essential cellular functions. Because KCNK family members are known to contribute to tumorigenesis in various types of cancer, we hypothesized that they might be differentially expressed in hepatocellular carcinoma (HCC) cells as compared to healthy tissue and serve as diagnostic or prognostic biomarkers. We tested this hypothesis through bioinformatic analyses of publicly available data for the expression of various KCNK subunits in HCC. We observed reduced expression of KCNK2, KCNK15, and KCNK17 in liver cancer, as well as overexpression of KCNK9, all of which correlated with a better prognosis for HCC patients per survival analyses. Moreover, ROC curves indicated that KCNK2, KCNK9, KCNK15, and KCNK17 levels could be used as a diagnostic biomarker for HCC. Finally, our western blot and qRT-PCR results were consistent with those obtained from bioinformatic analyses. Taken together, these results suggest that KCNK2, KCNK9, KCNK15, and KCNK17 could serve as potential diagnostic and prognostic biomarkers of HCC.

## INTRODUCTION

Liver cancer is one of the most common and deadly cancers worldwide with over 800,000 new cases and 780,000 deaths every year [1]. Hepatocellular carcinoma (HCC) is the most common pathological type of primary liver cancer, accounting for ~75-85% of cases. Despite great progress in the treatment of HCC in recent decades, HCC prognosis remains poor due to its high invasiveness and metastasis rates. Thus, novel prognostic and therapeutic targets for HCC merit further investigation.

Potassium ion channels are the most widely distributed and one of the most intricate protein complexes inside cells [2]. These ion channels, which maintain the resting membrane potential of excitable cells, function abnormally in cancer cells, including HCC cells [3–9]. KCNK channels (also known as K2P, for two-pore-domain potassium channels) are potassium-selective channels that tend to be constitutively open [10]. To date, 15 variants of KCNK subunits have been identified and are divided into five groups based on sequence homology and functional similarity (TWIK, TASK, THIK, TALK, and TREK channels) [11]. Most KCNKs behave as outward rectifiers under physiological K^+^ concentrations or behave in a nearly voltage-independent manner, helping to maintain the resting membrane potential [12]. Owing to this, they are essential for various cellular processes, such as metabolic regulation, apoptosis, and chemoperception [13]. KCNKs can also act as oncogenes in various cancers. For instance, some KCNK2 modulators inhibit apoptosis and promote proliferation in ovarian cancer [14]. The lncRNA KCNK15-AS1, which is downregulated in pancreatic cancer tissues, inhibited BxPC-3 cell invasiveness [15]. KCNK9 is upregulated in breast [16, 8] and colorectal [17] cancer, and increases tumor tolerance to hypoxia and a serum-free environment by inhibiting apoptosis. In the present study, we tested whether the expression of different KCNK proteins correlates with clinical parameters in HCC patients and whether such relationships have useful prognostic applications in HCC pathology.

## RESULTS

### Some KCNK mRNAs are differentially expressed between HCC and normal tissues

**Figure 1:**
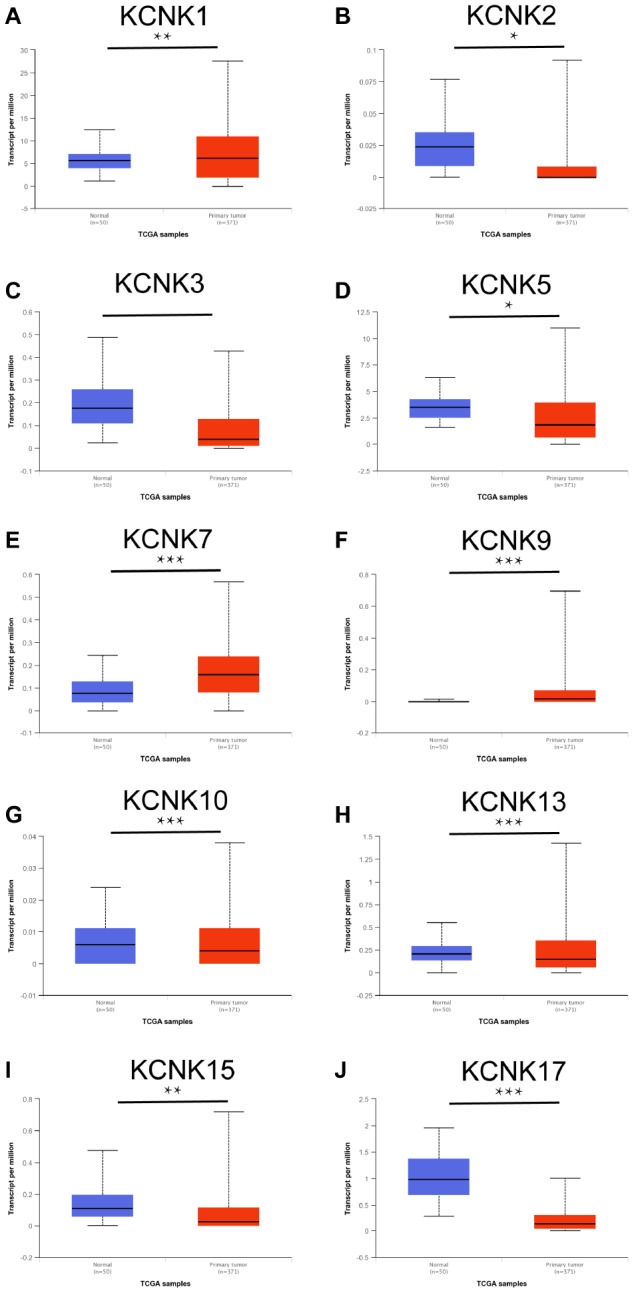
**KCNKs are differentially expressed between HCC and normal tissue (UALCAN).** KCNK1/7/9 mRNAs were overexpressed (**A**, **E**, **F**) while KCNK2/3/5/10/13/15/17 mRNAs were underexpressed (**B**, **C**, **G**, **H**, **I**, **J**) in primary HCC tissues compared to normal samples. *p<0.05, **p<0.01, *** p<0.001.

To understand the expression of KCNK gene family members in liver cancer tissues, we mined data from the UALCAN database (http://ualcan.path.uab.edu) and analyzed 15 KCNK mRNA levels in 371 HCC tissues and 50 matched non-tumor tissues. The top 10 genes with the largest differences between HCC and non-tumor tissues is listed in Figure 1. The data show that mRNA expression of KCNK1, KCNK7, and KCNK9 is upregulated in cancer tissues while KCNK2, KCNK3, KCNK5, KCNK10, KCNK13, KCNK15, and KCNK17 levels are decreased compared to controls. After screening by Bonferroni correction, the P values of KCNK7, KCNK9, KCNK10, KCNK13, and KCNK15 are less than the corrected P values (*p < 0.0033*). There was no difference in the expression of the remaining five KCNK genes between liver cancer cells and normal tissues (data not shown).

### Correlation between expression of 10 KCNKs and HCC patient clinicopathological characteristics

**Figure 2:**
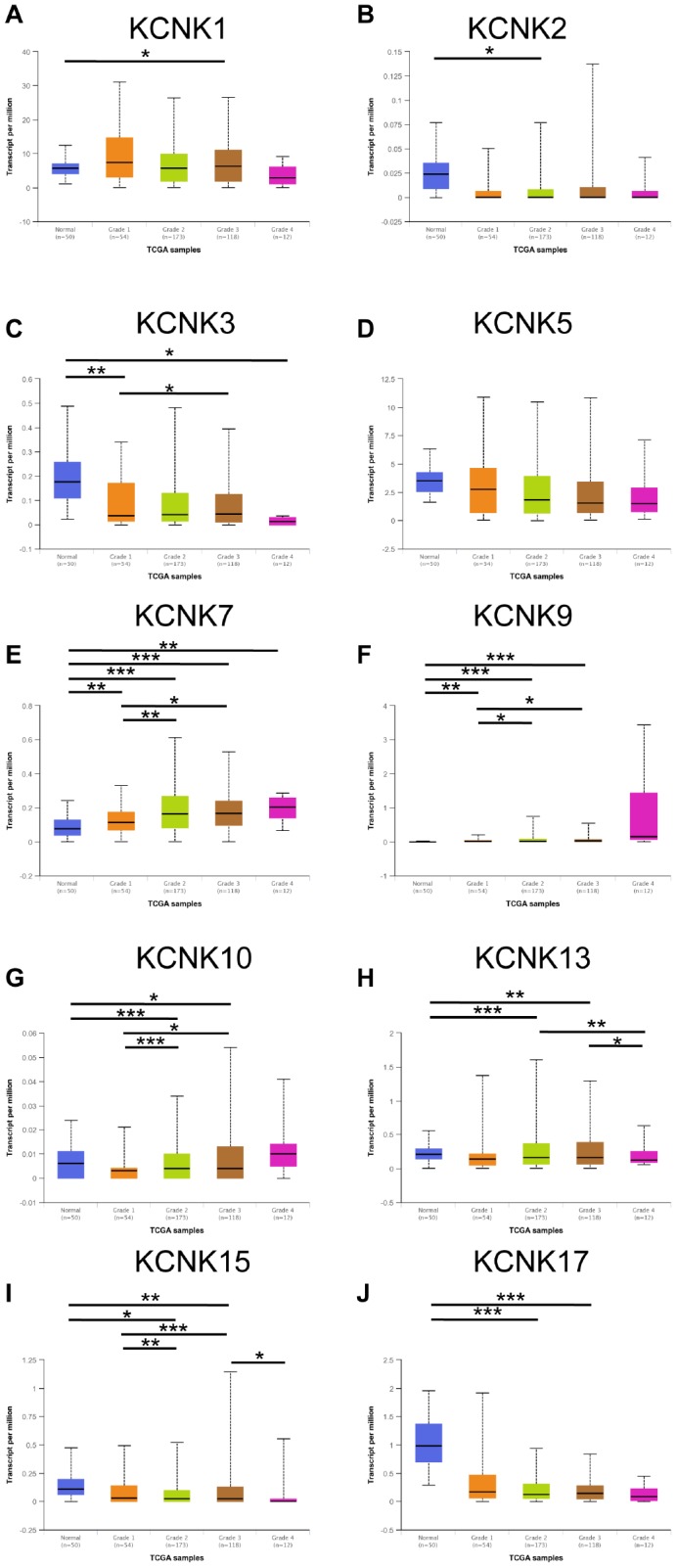
**Correlation between levels of 10 KCNKs and HCC patient clinicopathological characteristics.** The highest mRNA levels for KCNK7/9/10 were found in tumors of grade 4 (**E**, **F**, **G**) while the lowest mRNA levels of KCNK3/13/15/17 were found in grade 4 tumors (**C**, **H**, **I**, **J**). Expression of KCNK1/2/5 did not vary with tumor grade (**A**, **B**, **D**). *p<0.05, **p<0.01, ***p<0.001.

We next analyzed the relationship between the mRNA expression of 10 candidate KCNKs and the clinicopathological parameters of HCC patients using UALCAN (http://ualcan.path.uab.edu), including the patients’ individual tumor grades. The data showed that mRNA levels of KCNK7, KCNK9 and KCNK10 correlated inversely with tumor differentiation degree (Figure 2E–2G). On the other hand, mRNA levels of KCNK3, KCNK13, KCNK15, and KCNK17 correlated positively with tumor differentiation (Figure 2C, 2H, 2I, 2J). We did not observe differential expression of KCNK1, KCNK2, or KCNK5 for different tumor grades (Figure 2A, 2B, 2D). The results above suggested that the mRNA levels of some KCNKs correlate with clinicopathological parameters in HCC patients.

### KCNK1/2/9/17 levels are associated with HCC patient overall survival

**Figure 3:**
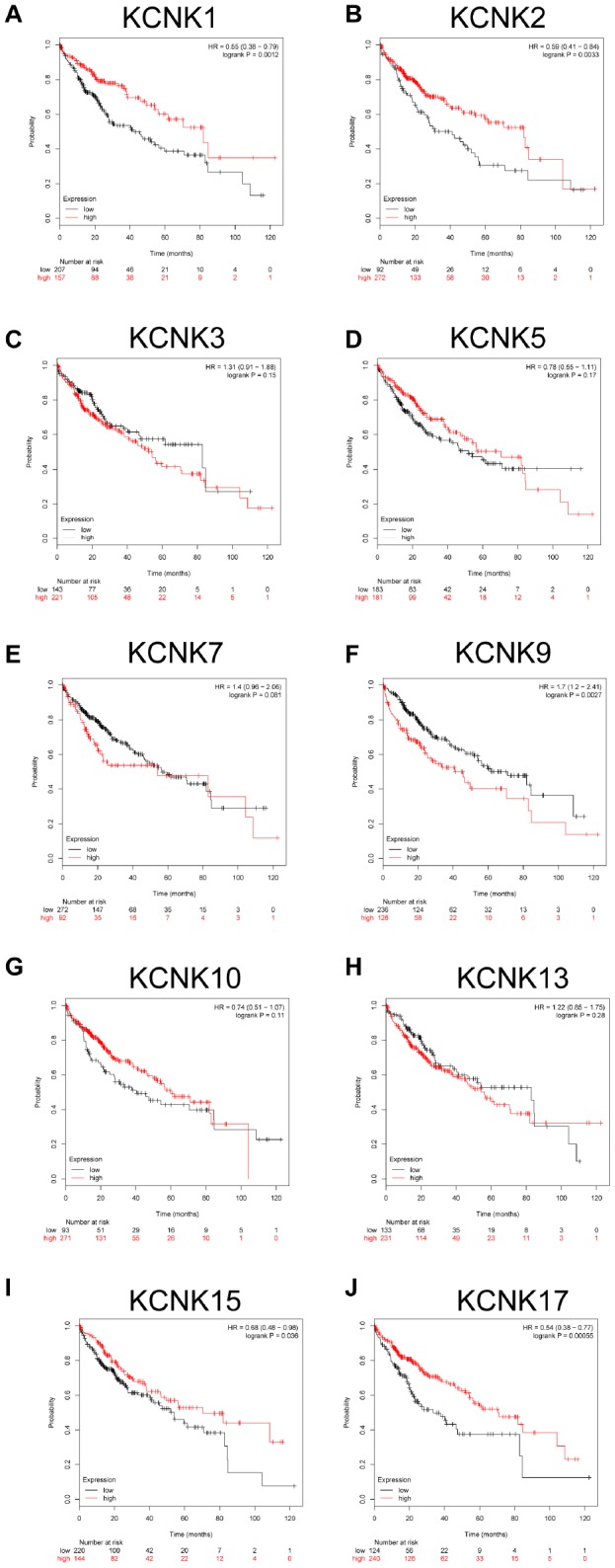
**Prognostic value of mRNA expression for distinct KCNKs in HCC (Kaplan-Meier Plotter).** KCNK9 mRNA levels correlated negatively (**F**) while KCNK1/2/15/17 mRNA levels correlated positively (**B**, **I**, **J**) with OS of HCC patients. KCNK7/10/13 mRNA levels showed no correlation with HCC patient prognosis (**E**, **G**, **H**).

We used a Kaplan-Meier plotter (http://kmplot.com/analysis/) to analyze the correlation between the mRNA levels of 10 candidate KCNKs and HCC patient prognosis. The results indicated that high expression of KCNK9 (HR=1.7, 95% CI: 1.2-2.41, and *p*=0.0027) was associated with a shorter overall survival (OS) rate in the HCC patients while low expression of KCNK2 (HR=0.59, 95% CI: 0.41-0.84, and *p*=0.0033), KCNK15(HR=0.68, 95% CI: 0.48-0.98, and *p*=0.036), and KCNK17(HR=0.54, 95% CI: 0.38-0.77, and *p*=0.00055) was associated with a longer OS rate. However, KCNK3, KCNK5, KCNK7, KCNK10, and KCNK13 mRNA expression did not correlate with HCC patient prognosis (Figure 3C–3D, 3G–3H). Interestingly, the result that high expression of KCNK1 was associated with a longer OS rate in HCC patients (HR=0.55, 95% CI: 0.38-0.79, and *p*=0.0012) was inconsistent with the difference analysis of the UALCAN database, which showed low KCNK1 expression in HCC. Bonferroni correction screening improved P values for KCNK1, KCNK2, KCNK9, and KCNK17.

### KCNK2/9/15/17 levels correlate with incidence of HCC

**Figure 4:**
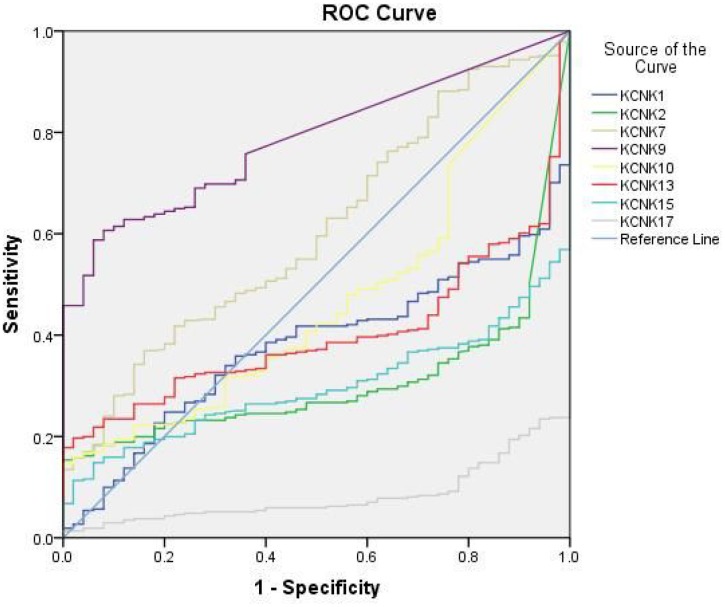
**Diagnostic value of eight KCNK candidate genes.** ROCs of KCNK1/2/7/9/10/13/15/17 levels in HCC, showing that elevated KCNK9 levels (**B**) and decreased KCNK2/15/17 levels (**A**, **C**, **D**) correlate with HCC incidence.

Based on the results of UALCAN and Kaplan-Meier analysis described above, KCNK1, KCNK2, KCNK7, KCNK9, KCNK10, KCNK13, KCNK15, and KCNK17 showed the greatest prognostic value for HCC among all the KCNKs examined. Therefore, we analyzed the diagnostic value of those 8 KCNKs in HCC by computing receiver operating characteristic (ROC) curves. The results showed that levels of KCNK2 (Area=0.308, 95% CI: 0.25-0.365, and *P*<0.0001), KCNK9 (Area=0.787, 95% CI: 0.737-0.836, and *P*<0.0001), KCNK15 (Area=0.298, 95% CI: 0.248-0.348, and *P*<0.0001), and KCNK17 (Area=0.083, 95% CI: 0.055-0.111, and *P*<0.0001) correlated with HCC incidence (Table 1). Detailed ROC results are shown in Figure 4. Our results suggest that KCNK2, KCNK9, KCNK15, and KCNK17 levels may be exploited as useful biomarkers to diagnose HCC and predict patient prognosis.

**Table 1:** The ROC test results of 8 candidate KCNK subunits.

**Gene**	**Area**	***P* value**	**95% Confidence Interval**	
			**Lower Bound**	**Upper Bound**
KCNK1	0.379623	0.00570	0.318462	0.440783
KCNK2	0.307682	0.00001	0.250332	0.365032
KCNK7	0.605984	0.01493	0.529307	0.682661
KCNK9	0.786523	0.00000	0.736836	0.83621
KCNK10	0.468652	0.47155	0.390298	0.547006
KCNK13	0.401078	0.02309	0.342249	0.459907
KCNK15	0.298248	0.00000	0.248176	0.34832
KCNK17	0.083019	0.00000	0.055487	0.110551

ROC: Receiver operating characteristics.

### Expression of KCNK2/9/15/17 correlates with HCC patient prognosis

**Figure 5:**
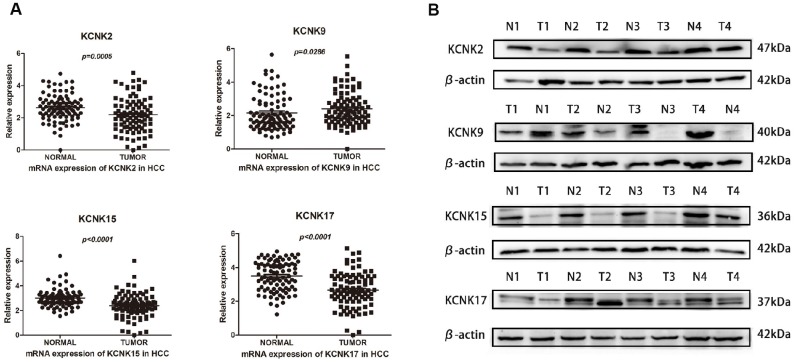
**Differential expression of KCNK2/9/15/17 mRNA in surgically removed HCC tissues.** KCNK9 mRNA levels are elevated while KCNK2, KCNK15, and KCNK17 mRNA levels are decreased in HCC tissues compared with normal tissues as measured by qRT-PCR (**A**). KCNK9 protein levels are increased while KCNK2/15/17 protein levels are decreased in HCC tissues compared to controls, as measured by Western Blot (**B**).

**Figure 6:**
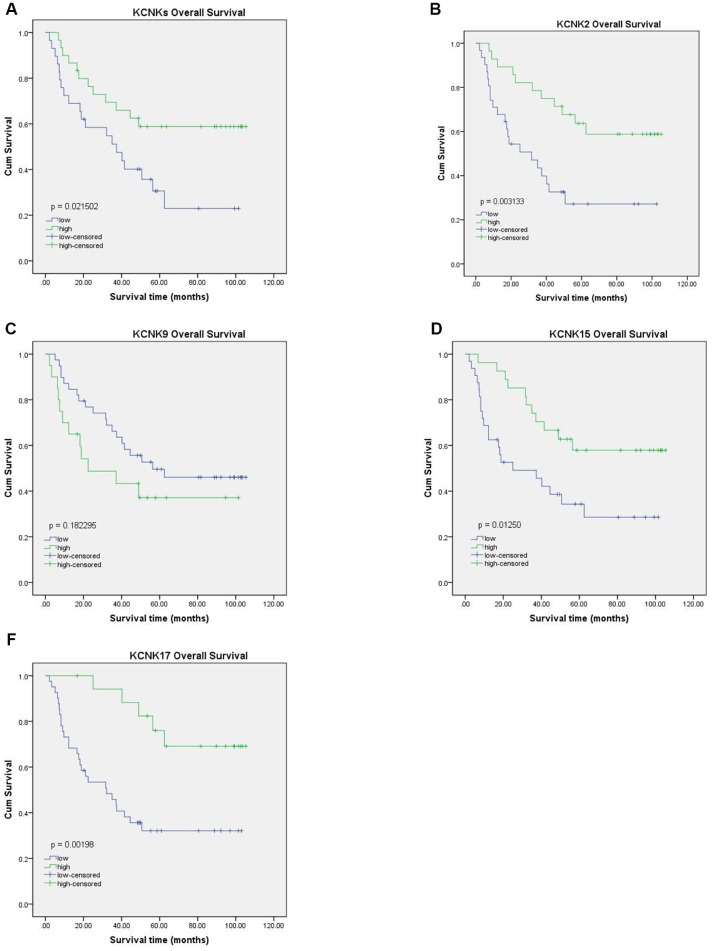
**Prognostic value of KCNK2, KCNK9, KCNK15, and KCNK17 mRNA levels in HCC patients.** Increased KCNK2, KCNK15, and KCNK17 mRNA levels correlated with favorable OS of HCC patients (**B**, **D**, **F**). KCNK9 mRNA levels showed no correlation with HCC patient prognosis (**C**). Mean KCNK2/9/15/17 levels showed prognostic value in patients with HCC, with higher levels suggesting better prognosis (**A**).

To confirm our conclusions above, we used qRT-PCR to measure the mRNA levels of KCNK2, KCNK9, KCNK15, and KCNK17 in 90 pairs of HCC specimens and matched non-tumor specimens, which were surgically removed from HCC patients. We found that KCNK9 (*p* = 0.0286) is upregulated in HCC tissues compared with normal controls while KCNK2 (*p* = 0.0005), KCNK15 (*p* < 0.0001), and KCNK17 (*p* < 0.0001) are downregulated (Figure 5A), which is consistent with our analyses using data from the UALCAN database. Next, we used Western Blot to measure the protein levels of KCNK2, KCNK9, KCNK15, and KCNK17 in four pairs of HCC tissues and matched non-tumor tissues. We found that KCNK9 protein levels were elevated while those of KCNK2, KCNK15, and KCNK17 were lower in HCC tissues than in controls (Figure 5B). We used Kaplan-Meier plots to test the prognostic value of these four KCNKs and found that high levels of KCNK2 (*p* = 0.003133; Figure 6B), KCNK15 (*p* = 0.0125; Figure 6D), or KCNK17 (*p* = 0.00198; Figure 6E) correlated with improved patient prognosis. On the other hand, KCNK9 (*p* = 0.182295; Figure 6C) had no prognostic value. Of note, the mean levels of KNCK2/9/15/17 also correlated with HCC patient prognosis, with higher levels suggesting better prognosis (*p* = 0.021502; Figure 6A).

### Functional enrichment analysis

**Figure 7:**
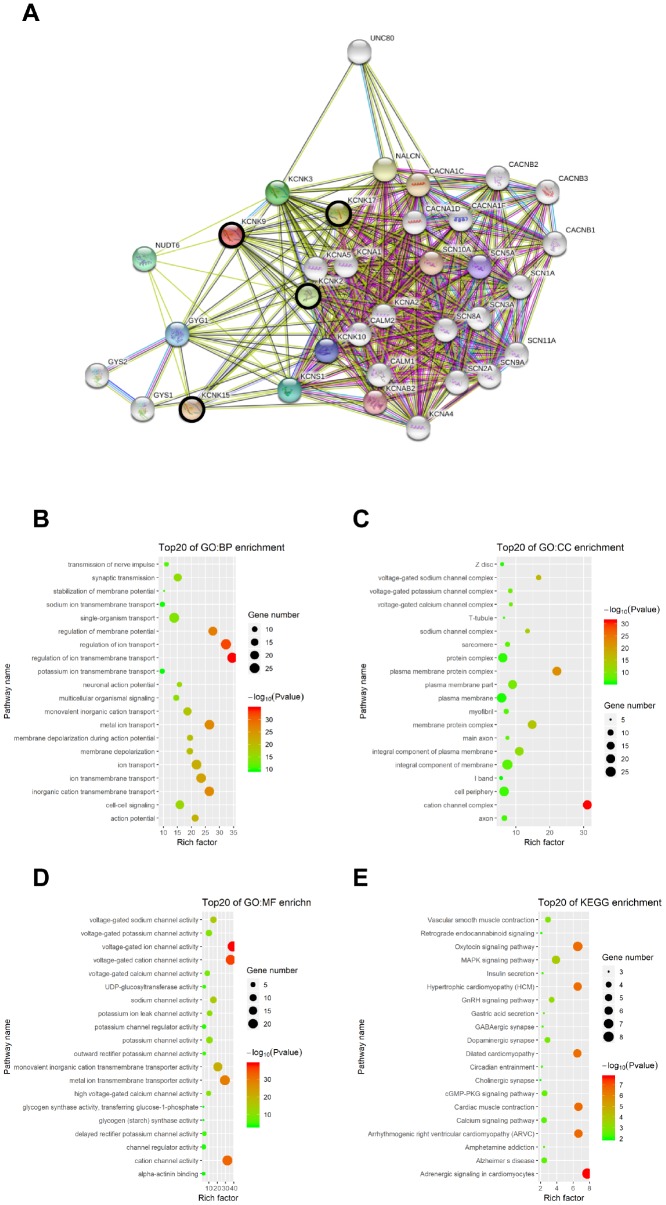
**Functional enrichment analysis of KCNKs through STRING database.** We analyzed the network of KCNK mutations and their 50 most frequently altered neighboring genes; sodium channels SCN1A, SCN2A, SCN3A, SCN5A, SCN8A, SCN9A, SCN10A, and SCN11A, and calcium channel subunits CACNA1C, CACNA1D, CACNA1F, CACNB1, CACNB2, and CACNB3 were associated with KCNK mutations (**A**). GO and KEGG functional enrichment analysis showed that voltage-gated ion transport and ion transmembrane transport function, action channel activity function, adrenergic signaling pathway, oxytocin signaling pathway, and hypertrophic cardiomyopathy were compromised by mutations in the KCNK2/9/15/17 genes (**B**, **C**, **D**, **E**).

We used the STRING database to elucidate the 50 most relevant neighboring genes and their functional characteristics associated with KCNK2/9/15/17 mutations through enrichment analysis of the GO and KEGG pathways. Our results showed that sodium channels (SCN1A, SCN2A, SCN3A, SCN5A, SCN8A, SCN9A, SCN10A, and SCN11A) and calcium channel subunits (CACNA1C, CACNA1D, CACNA1F, CACNB1, CACNB2, and CACNB3) were associated with KCNK2/9/15/17 subunit mutations (Figure 7A). Moreover, six GO items and six KEGG pathways were enriched in combinations of those four genes (P < 0.05; Figure 7B–7E), including in the regulation of voltage-gated ion transport and ion transmembrane transport function, action channel activity function, adrenergic signaling pathway, oxytocin signaling pathway, and hypertrophic cardiomyopathy.

## DISCUSSION

Hepatocellular carcinoma remains one of the most lethal malignancies worldwide because of its complex molecular and cellular heterogeneity, and its incidence is increasing [18]. Although more than 200 genes related to the proliferation, invasion and metastasis of liver cancer have been reported, the number of specific prognostic biomarkers and therapeutic targets remains small [19]. Comprehensive screening of molecular biomarkers for liver cancer may improve prognosis and reduce mortality in HCC.

Ion channels are transmembrane proteins that regulate the flow of ions across biofilms and participate in many cellular functions. Overington et al. reported a variety of drugs targeting ion channels (~13% of all drugs), which are used to treat many human diseases, including cardiovascular and nervous system diseases [20]. Recent studies report that ion channels promote HCC incidence and metastasis of HCC cells [21–25]. KCNK channels are potassium-selective channels that behave as outward rectifiers under physiological K^+^ concentrations or behave in a nearly voltage-independent manner, helping to maintain the cell’s resting membrane potential.

In our study, we found through bioinformatics analyses that KCNK2, KCNK9, KCNK15, and KCNK17 mRNA and protein levels can be used as diagnostic biomarkers in HCC and predict patient prognosis. We also analyzed GO function, KEGG pathway, and protein-protein relationships among these four KCNKs and correlated genes to predict their function. Our results showed that KCNK2, KCNK9, KCNK15, and KCNK17 participate in transmembrane ion transport, voltage-gated ion channel activity, and adrenergic cardiomyocyte signaling.

KCNK2 (also known as TREK-1) is a member of the two-pore-domain K^+^ channel family (K2P) [26], which is responsible for maintaining neuronal resting membrane potential and the duration of action potentials, also participating in neurotransmitter release [27] among other biological functions [28, 29]. KCNK2 is overexpressed in prostate [30] and epithelial ovarian [14] cancer, among other cancer types. In the present study, we found that KCNK2 is underexpressed in HCC, which is accompanied by poor OS. Therefore, further investigation is warranted to test whether KCNK2 can be targeted for therapeutic benefit.

KCNK9 belongs to the TWIK-related acid sensitive K^+^ channel (task-3) and the bilateral potassium channel families [31]. Similar to KCNK2, KCNK9 is distributed in various human tissues where it is involved in many physiological activities. Unsurprisingly, its disruption can contribute to the occurrence and development of many diseases, including cancer. For example, mutations in the KCNK9 gene can lead to KCNK9 imprinting syndrome [32]. Furthermore, KCNK9 is overexpressed in breast cancer [8], rectal cancer [17], melanoma [33], and adrenal cortical adenocarcinoma [34]. Pocsai et al. reported that KCNK9 is overexpressed in the mitochondria of melanoma cells, perhaps sustaining the uncontrolled growth of tumors. Although the mechanisms underlying its oncogenic activity remain to been elucidated, the overexpression that we uncovered here for KCNK9 in HCC might serve as an effective diagnostic biomarker.

KCNK15 is a member of the superfamily of potassium channel proteins containing two pore-forming P domains and requiring other non-pore-forming proteins to be active [35]. While KCNK17 channels expressed in human heart atrial tissue [36] represent potential therapeutic targets to treat atrial and ventricular arrhythmias [37], to the best of our knowledge, our study is the first to report dysregulated expression of KCNK15 and KCNK17 mRNA and protein in HCC tissues.

Our study suffers from various limitations and its conclusions should be further tested through multicenter, large-sample studies using patient data from multiple databases rather than a single database. Similarly, further studies are needed to investigate the molecular mechanisms underlying KCNK oncogenic effects on HCC, which our study did not address. Despite these limitations, our study is the first to report differential expression of KCNKs in HCC and their potential diagnostic and prognostic value. We observed decreased KCNK2, KCNK15, and KCNK17 levels in HCC tissues, which correlated with unsatisfactory patient prognosis. In addition, KCNK9 levels were increased in HCC. Taken together, our data highlight KCNK2, KCNK9, KCNK15, and KCNK17 as potential diagnostic and prognostic biomarkers as well as potential targets to treat HCC.

## MATERIALS AND METHODS

### Data preparation

Data concerning the mRNA expression profile and corresponding clinical information from the KCNK family members across the HCC samples and normal tissue, as well as the tumor subgroup, were obtained from the Cancer Genome Atlas (TCGA) database, which was accessed through the University of California Santa Cruz Xena data hub (UCSC Xena: https://xena.ucsc.edu/, retrieved June 21,2018). The platform contained 371 HCC tissues and 50 adjacent non-tumor liver tissues. The data from the TCGA are publicly available and open-access, and this study follows the TCGA data access policy and published guidelines.

### Patients and specimens

All clinical specimens used to measure protein and mRNA levels of KCNK2, KCNK9, KCNK15, and KCNK17 were collected from the third Affiliated Hospital of Sun Yat-Sen University, Guangzhou, China. The protocol of this study was approved by the ethics committee of the third Affiliated Hospital of Sun Yat-Sen University. Specimens were collected in accordance with legal regulations, and informed consent was obtained from each patient.

### Quantitative reverse transcription-PCR (qRT-PCR)

Total RNA was extracted from 90 pairs of HCC patient tissues using Trizol reagent (Life Technologies, CA, USA). We performed qRT-PCR analyses using a real-time PCR 480 system and SYBR-green PCR Master Mix. The PCR procedure was set as 95 °C for 5 min followed by 45 cycles of 95 °C for 10 s, 60 °C for 20 s, and 72 °C for 20 s. A 2-Δ CT value was used to determine relative mRNA expression for KCNK2, KCNK9, KCNK15, and KCNK17. For qRT-PCR, the following primer sequences for KCNK family members were used: KCNK2 Forward 5′ CTGACCAGCGAGAGGGATGT 3′ Reverse 5′CCTATGGCTATGCCTAAGGTTATTT 3′, KCNK9 Forward 5′ATCACACATCATAGCCTGCTTTTG 3′, Reverse 5′CCATGACACATCAGGGATAAGAACT 3′, KCNK15 Forward 5′CAGAACCCTGCTCCCTCTTAC3′, Reverse 5′ACACCTCGGGCTTTGTCTCT3′, KCNK17 Forward 5′ACGCCAGGGAGGGTATGTT 3′, and Reverse 5′AGAAGGTTCCAGATGCTGTATGA 3′.

### Western blot analysis

Protein was extracted from four pairs of tissue from HCC patients using RIPA buffer (Pierce, Rockford, USA). The tissue protein content was quantitatively analyzed using the BCA protein Assay kit (Pierce). Equal amounts of proteins (30 ug) were separated using 10% SDS polyacrylamide gels and then transferred to a PDVF membrane. The PDVF membrane was blocked with 5% skim milk diluted with Tris-buffered saline containing Tween 20 (TBS-T) for 90 min and the incubated with primary anti-KCNK2 antibodies (1:200 dilution, Sabin), KCNK9 (1:1000 dilution, Abcam), KCNK15 antibodies (1:1000 dilution, Absin), or KCNK17 antibodies (1:800 dilution, Absin) at 4 °C overnight followed by incubation with secondary goat anti-rabbit IgG (1:1000 dilution, abclonal) at 37 °C for 1 h. Immunodetection was performed through chemiluminescence (ECL, Guangzhou, China), and *β*-actin (1:1000 dilution, abclonal) or GAPDH (1:2000 dilution, abclonal) was used as loading control.

### UALCAN analysis

UALCAN (http://ualcan.path.uab.edu) is an online in-depth analysis of gene expression variations across major cancer types and normal tissues, as well as tumor subgroups, and its aim is to identify tumor subgroup specific candidate biomarkers [38]. In this study, we analyzed the mRNA levels of 15 KCNKs in HCC tissues. Differences in transcriptional expression were compared with *t*-test, while the relationships between KCNKs mRNA levels and different individual tumor grades were analyzed using one-way ANOVA. Bonferroni correction was applied to establish significance. Therefore *P* < 0.0033 was considered to be statically significant.

### Survival analysis and diagnostic prediction

The Kaplan-Meier survival analysis was used to evaluate the prognostic value of distinct expression profiles among KCNKs family members in HCC patients. The HCC patients were divided into high and low mRNA expression groups by the best cut-off value obtained from receiver operating characteristic (ROC) curves. Bonferroni correction was applied to establish significance and *P* value < 0.0033 was considered to be statistically significant. In addition, ROC curves were computed to evaluate the predictive power of the eight candidate KCNKs in HCC diagnosis.

### Functional enrichment analysis

The Gene Ontology (GO) and Kyoto Encyclopedia of Genes and Genomes (KEGG) pathway enrichment analyses of the KCNK2, KCNK9, KCNK15, and KCNK17 protein coding genes were conducted using the STRING database (https://string-db.org/).

### Statistical methods

GraphPad Prism software version 5.0 was used to analyze KCNK2, KCNK9, KCNK15, and KCNK17 mRNA levels in HCC tissues compared to matched normal liver tissue controls, and differences in transcriptional expression were compared using *t*-test. Kaplan-Meier survival curves and ROC curves were plotted using the SPSS software version 22.0.
